# ICCTUS score - Inspiration, Circulation and Consciousness predicting the Threat for an Unfavorable Outcome after SAH

**DOI:** 10.3389/fmed.2026.1743482

**Published:** 2026-01-26

**Authors:** Elena Kurz, Verena Fassl, Alicia Schulze, Darius Kalasauskas, Florian Ringel, Axel Neulen

**Affiliations:** 1 Department of Neurosurgery, University Medical Center of the Johannes Gutenberg-University of Mainz, Mainz, Germany; 2 Institute of Medical Biostatistics, Epidemiology and Informatics, University Medical Center of the Johannes Gutenberg-University of Mainz, Mainz, Germany; 3 Department of Neurosurgery, LMU University Hospital, Munich, Germany

**Keywords:** clinical study, ICCTUS score, outcome, sequential organ failure assessment score, subarachnoid Hemorrhage

## Abstract

**Background:**

We previously developed a novel score derived from the Sequential Organ Failure Assessment score and demonstrated its ability to predict delayed cerebral ischemia-associated infarctions following spontaneous subarachnoid hemorrhage. In the current study, we investigated whether the new score (ICCTUS score) can predict neurological outcome.

**Methods:**

We retrospectively evaluated all SAH patients in our neurosurgical ICU during a 10-year period. Patients were included if clinical data were available to determine SOFA and ICCTUS scores. Outcome was objectified by the modified Rankin Scale (mRS) after 6 months. Every parameter of the SOFA score was graded for its predictive value and combinations were tested using ROC analysis.

**Results:**

430 patients fulfilled the inclusion criteria (68.14% female, mean age: 56.8 ± 12.5 years). Median SOFA and ICCTUS scores were 5. The SOFA score had an AUC of 0.76 for prediction of unfavorable outcome. In comparison, the WFNS achieved an AUC of 0.71, and the HH an AUC of 0.64. For the ICCTUS score, which is based exclusively on the subscores rating the central nervous system, the cardiovascular system, and the respiratory system the AUC was at 0.8 with a sensitivity of 0.74, a specificity of 0.74, a PPV of 0.83 and a NPV of 0.62. The Youden index was 0.48 (cut-off ≥3 points).

**Conclusion:**

The ICCTUS score was at least equal or superior to the established scores in predicting unfavorable outcome after SAH. The score could be implemented as an additional tool in multimodal diagnostics to identify patients at high risk.

## Introduction

Spontaneous subarachnoid hemorrhage accounts for approximately 5–10% of all strokes and is associated with high rates of mortality and long-term disability (2, 3). Since neurological deterioration can occur delayed after the bleeding event (4) identifying patients at high risk of unfavorable outcome early is important in order to provide appropriate monitoring resources for these cases.

The most important factor determining functional outcome after SAH is brain injury, which occurs in two phases (3–6): Early brain injury, which is the direct consequence of bleeding and transient global cerebral ischemia, occurs in the first 72 h after SAH; delayed cerebral ischemia (7) occurs between 4 and 21 days post-SAH and is caused by different complex pathophysiological mechanisms, which are essentially initiated by the initial bleeding and EBI (7). Consequently, clinical scores which represent the degree of EBI like the Hunt & Hess score (H&H) (8) or the world federation of neurosurgical societies score (WFNS) (9) were shown to correlate with functional outcome.

However, besides cerebral pathophysiological events, systemic processes initiated by the hemorrhage lead to peripheral organ dysfunctions with varying degrees of severity in a large subgroup of SAH patients, most importantly neurogenic cardiomyopathy and neurogenic pulmonary edema (10–13). Besides direct effects on functional outcome, clinical and experimental studies indicate that neurogenic cardiomyopathy and neurogenic pulmonary edema also contribute to brain injury, especially DCI but also EBI, by impairing cerebral blood and oxygen supply (11, 14, 15).

The Sequential Organ Failure Assessment (SOFA) is widely used in intensive care medicine to monitor organ dysfunction (16, 17) and was shown to predict mortality in sepsis patients (18). It assesses organ systems including cardiovascular function, respiratory function, neurological status (level of consciousness), hepatic function, renal function, and coagulation parameters. Furthermore, it has also been reported to correlate with in hospital mortality after SAH (15). In a previous study we found that the highest SOFA score collected during the first 48 h after SAH predicted the risk for DCI-associated infarctions in SAH patients (19). In accordance with the current pathophysiological concepts, the most robust parameters were the categories GCS, reflecting brain injury, blood pressure reflecting cardiac function, and respiratory function. Based on these categories we developed a novel score as a further development from the SOFA score (Table 1A), to reflect the pathophysiology after SAH. The novel score was superior in predicting DCI-associated infarctions compared to H&H, WFNS, and the SOFA score (19).

**Table 1:** (A) SOCA score- included parameters and scorings, (B) ICCTUS score - included parameters and scorings, (C) Flowchart depicting inclusion criteria and creation of the patient cohort.

(A) SOFA score						
Points	Creatinine (mg/dl)	Bilirubin (mg/dl)	Number of thrombocytes (x103/μl)	Horovitz quotient (PaO_2_/FiO_2,_ mmHg)	MAP	GCS
0	< 1.2	< 1.2	≥ 150	≥ 400	≥ 70 mmHg	15
1	1.2–1.9	1.2–1.9	<150	<400	<70 mmHg	14–13
2	2.0–3.4	2.0–5.9	<100	<300	Dopamin(≤5 μg/kg/min) OR Dobutamin	12–10
3	3.5–4.9	6.0–11.9	<50	<200 + ventilation	Dopamin(>5 μg/kg/min) OR Adrenalin(≤0.1 μg/kg/min) OR Noradrenalin(≤0.1 μg/kg/min)	9–6
4	> 5	> 12.0	<20	<100 + ventilation	Dopamin(>15 μg/kg/min) OR Adrenalin(>0.1 μg/kg/min) OR Noradrenalin(>0.1 μg/kg/min)	< 6

(B) ICCTUS score			
Points	Horovitz quotient (PaO_2_/FiO_2,_ mmHg)	MAP	GCS
0	≥ 400	≥ 70 mmHg	15
1	<400	<70 mmHg	14–13
2	<300	Dopamin(≤5 μg/kg/min) OR Dobutamin	12–10
3	<200 + ventilation	Dopamin(>5 μg/kg/min) OR Adrenalin(≤0.1 μg/kg/min) OR Noradrenalin(≤0.1 μg/kg/min)	9–6
4	<100 + ventilation	Dopamin(>15 μg/kg/min) OR Adrenalin(>0.1 μg/kg/min) OR Noradrenalin(>0.1 μg/kg/min)	< 6

(C) Flowchart
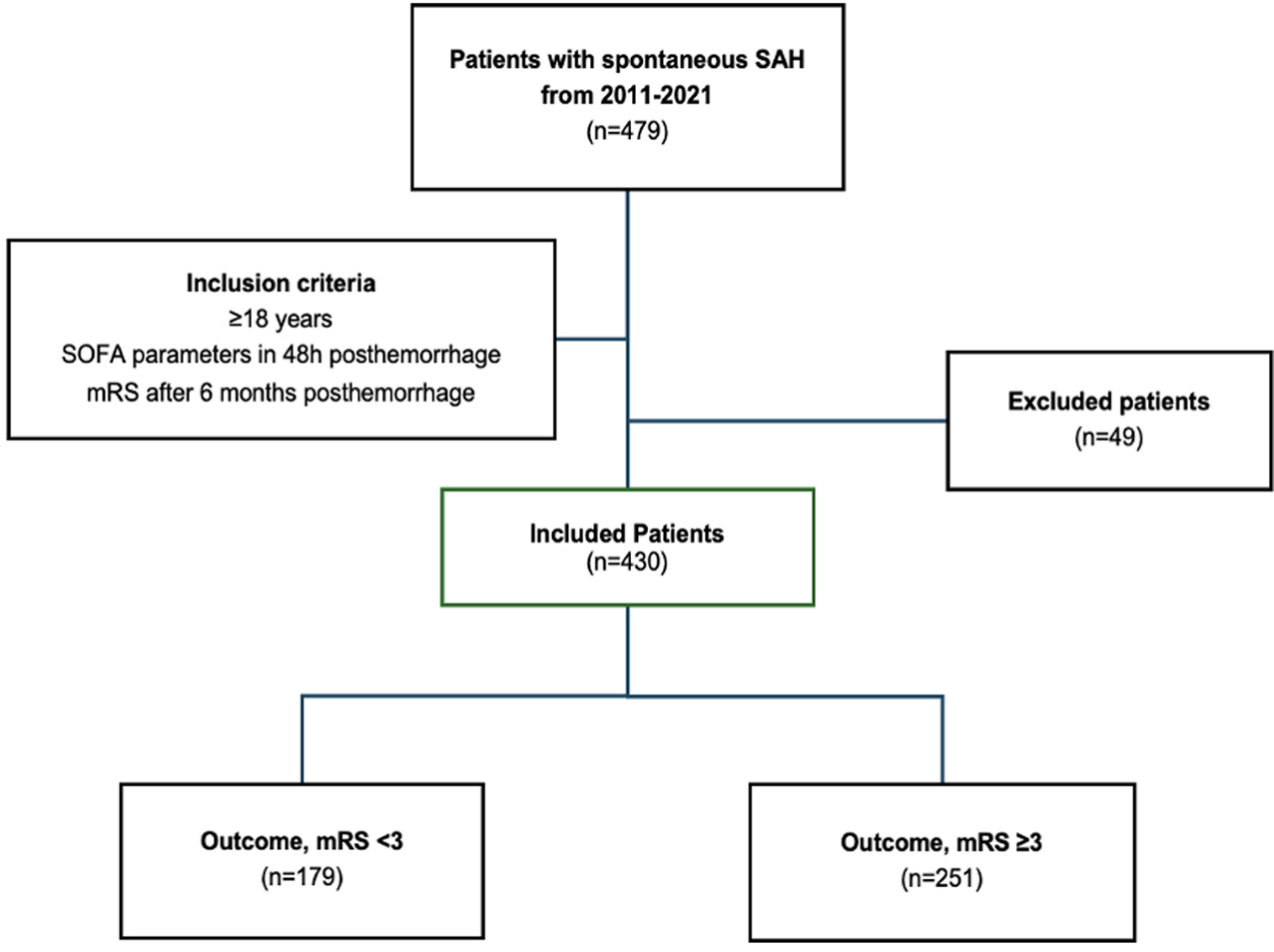

GCS Glasgow Coma Scale; MAP Mean Arterial Pressure.

Since the occurrence of DCI and DCI-associated infarctions is closely associated with unfavorable functional outcome, we hypothesized that our novel score would also predict functional outcome after SAH. The aim of our study was therefore to test whether our novel score, hereinafter referred to as ICCTUS Score (*I*nspiration, *C*irculation and *C*onsciousness predicting the *T*hreat for an *U*nfavorable Outcome after *S*AH) (Table 1B) predicts unfavorable neurological outcome after SAH and to compare its efficacy to the established H&H, WFNS, and SOFA scores.

## Materials and methods

### Study design and ethical approval

This investigation was conducted as a retrospective observational cohort study and was approved by the Ethics Committee of the Rhineland-Palatinate State Chamber of Physicians. The study adhered to the principles of the Declaration of Helsinki and its later amendments. Due to its retrospective nature and anonymized data, informed consent was waived.

### Setting and participants

We retrospectively included all adult patients (≥18 years) admitted to the neurosurgical intensive care unit (ICU) of our tertiary care center with aneurysmal subarachnoid hemorrhage (20) over 10 years (January 2011 to June 2021). Clinical management during the study period followed standardized departmental protocols (21, 22).

The inclusion criteria were (Table 1C):

1. Complete data were available to calculate the Sequential Organ Failure Assessment (SOFA) score on days 1 and 2 following SAH.
2. Availability of the modified Rankin Scale (mRS) from a follow-up visit, to which patients were invited 6 months post-SAH.

### Data sources and measurement

SOFA score parameters were extracted from electronic health records.

The following baseline characteristics were recorded: age, sex, comorbidities, aneurysm location, treatment modality (clipping or endovascular intervention), H&H grade, Fisher score, WFNS grade, and SOFA score. Functional outcome was assessed using the mRS at 4–8 months post-hemorrhage.

### Bias and study size

Due to the retrospective design, no formal sample size calculation was conducted; all eligible patients during the study period were included.

### Statistical analysis

Categorical variables were summarized using absolute and relative frequencies, while continuous variables were reported as means with standard deviations (23). Logistic regression analysis was used to evaluate associations between clinical predictors, and 6-month functional outcomes.

The Box–Tidwell test indicated that the linearity-in-the-logit assumption was met for all continuous predictors. The six SOFA components, being categorical, were individually assessed for their ability to predict functional neurological outcome after 6 months using the mRS.

All possible combination of the three most valuable SOFA score components were evaluated using the Akaike Information Criterion (AIC) to identify the most parsimonious and predictive models. The combinations yielding the lowest AICs were selected as simplified candidate models. Based on individual predictors with the highest AUC values, these combinations demonstrated superior model fit. Receiver operating characteristic (ROC) curve analysis was conducted for each model, and AUC values were used to quantify predictive performance. Comparisons between the newly developed models and established scoring systems were performed using the DeLong test. The optimal threshold for DCI prediction in each model was determined using the Youden Index. All analyses were carried out using R statistical software (version 4.4.1; R Core Team, 2021) (21).

## Results

A total of 479 patients with SAH treated at the Department of Neurosurgery, University Medical Center Mainz, Germany, between January 2011 and June 2021 were screened for inclusion (Table 1C). Of these, 430 patients met the inclusion criteria and were enrolled in the final analysis. The demographics and clinical characteristics of the cohort are representative and consistent with previously published descriptions of SAH populations. Further details are provided in Table 2.

**Table 2:** (A) Table of demographics and scores of the study population and the subgroups of patients with good and a poor outcome, (B) Comparison between good and poor outcomes, *p*-values and odds ratio.

(A) Demographics			
Parameters	General	mRS <3	mRS ≥3
Number, *n* (%)	430	179 (41.6)	251 (58.4)
Sex			
Male, *n* (%)	137 (31.9)	63 (35.2)	74 (29.5)
Female, *n* (%)	293 (68.1)	116 (64.8)	177 (70.5)
Age			
Mean (SD, years)	56.8 (12.5)	53.9 (12.3)	59.6 (12.1)
Endovascular treatment	238 (55.4)	99 (55.3)	139 (55.4)
Surgery	148 (34.4)	49 (27.4)	99 (39.4)
No intervention (negative DSA)	44 (10.2)	31 (17.3)	13 (5.2)
Hunt & Hess score Median	3	2	3
Grade 1 *n*, (%)	82 (19.1)	48 (26.8)	24 (9.6)
Grade 2 *n*, (%)	118 (27.4)	75 (41.9)	43 (17.1)
Grade 3 *n*, (%)	91 (21.2)	39 (21.8)	52 (20.7)
Grade 4 *n*, (%)	43 (10.0)	10 (5.6)	33 (13.2)
Grade 5 *n*, (%)	96 (22.3)	7 (3.9)	89 (35.5)
WFNS score median	3	4	2
Grade 1 *n*, (%)	78 (18.1)	49 (27.4)	29 (11.6)
Grade 2 *n*, (%)	102 (23.7)	66 (36.9)	36 (14.2)
Grade 3 *n*, (%)	85 (19.8)	37 (20.7)	48 (19.1)
Grade 4 *n*, (%)	69 (16.1)	20 (11.2)	49 (19.5)
Grade 5 *n*, (%)	96 (22.3)	7 (3.9)	89 (35.5)
Fisher score median	4	3	4
Grade 1 *n*, (%)	6 (1.4)	6 (3.4)	0 (0.0)
Grade 2 *n*, (%)	19 (4.4)	13 (7.3)	6 (2.4)
Grade 3 *n*, (%)	145 (33.7)	91 (21.2)	132 (52.6)
Grade 4 *n*, (%)	260 (60.5)	69 (38.6)	191 (76.1)
SOFA score median	5	3	7
0–5 points *n*, (%)	240 (55.8)	146 (81.6)	94 (37.5)
6–10 points *n*, (%)	179 (41.7)	33 (18.4)	146 (58.2)
11–15 points *n*, (%)	11 (2.6)	0 (0.0)	11 (4.4)
16–27 points *n*, (%)	0 (0.0)	0 (0.0)	0 (0.0)

(B) Comparison			
Parameters	mRS <3	mRS ≥3	*p*-value, Odds ratio
Age	53.3	59.6	*p* < 0.0001, 1.03
Sex			
Male	63	74	*p* = 0.24
Female	116	177	
Hypertension	59	112	*p* = 0.09
Smoking	49	61	*p* = 0.67
WFNS score	2	4	*p* < 0.001, 1.73
HH score	2	3	*p* < 0.0001, 1.74
Fisher score	3	4	*p* < 0.0001, 1.4
SOFA score	3	7	*p* < 0.0001, 1.5
Therapy			
Clipping	49	99	*p* = 0.13
Coiling	99	139	
No intervention	13	31	
EVD	69	179	*p* < 0.001, 5.3
Vasospasm	28	46	*p* < 0.0001, 2.1

EVD, external ventricular drainage; MCA, middle cerebral artery; ICA, internal cerebral artery; SD, Standard deviation; SOFA, Sequential Organ Failure Assessment score; WFNS, Word Federation of Neurosurgical Societies score.

The median ICCTUS score was 5. The median Sequential Organ Failure Assessment (SOFA) score was 5, the median Hunt and Hess (H&H) score was 3, the median Fisher score was 4, and the median World Federation of Neurosurgical Societies (WFNS) score was 3. Neurological outcome, assessed using the modified Rankin Scale (mRS) at 6 months post-hemorrhage, yielded a median score of 3. In total, 179 patients had a favorable outcome (mRS < 3), while 251 patients were classified as having an unfavorable outcome (mRS ≥ 3).

There was no significant difference between the subgroup of patients with a good outcome (mRS < 3) and the subgroup of patients with a bad outcome (mRS ≥ 3) regarding sex, arterial hypertension, and smoking. However, a significant difference was noted in age (*p* < 0.001), the evaluated established scores (SOFA: *p* < 0.001, HH: *p* < 0.001, WFNS: *p* < 0.001, Fisher: *p* < 0.001), vasospasm (*p* = 0.001), the need for an EVD (*p* < 0.001) and the kind of aneurysm occlusion (*p* < 0.001) (Table 2B).

### Evaluation of the predictive value of the ICCTUS score

Given the organ systems most frequently affected during the course of subarachnoid hemorrhage (10, 20)—namely the heart, lungs, and central nervous system—we developed a composite score based on parameters that specifically reflect these systems in a previous study (19). We focused on the Glasgow Coma Scale (GCS), reflecting neurological status; the Horovitz ratio (PaO₂/FiO₂), indicative of pulmonary function; and mean arterial pressure (MAP), as a surrogate for cardiovascular performance. Each parameter was graded in accordance with the SOFA scoring system.

The selected parameters are derived from established scoring systems such as the Sequential Organ Failure Assessment (SOFA) score, which has previously been shown in literature to correlate with in hospital mortality following SAH (15, 23). However, the SOFA score includes several organ systems that are of limited relevance in the context of SAH.

In our previous study, we demonstrated that our new score could predict the occurrence of delayed cerebral ischemia (DCI) associated infarctions after SAH. Since DCI is an important factor for neurological outcome, we applied this score to our current cohort to assess its predictive capacity for outcome following SAH.

In addition to evaluating the original three-parameter score, we analyzed the Glasgow Coma Scale (GCS) separately, as it emerged as the strongest individual predictor among the SOFA components.

**Figure 1:**
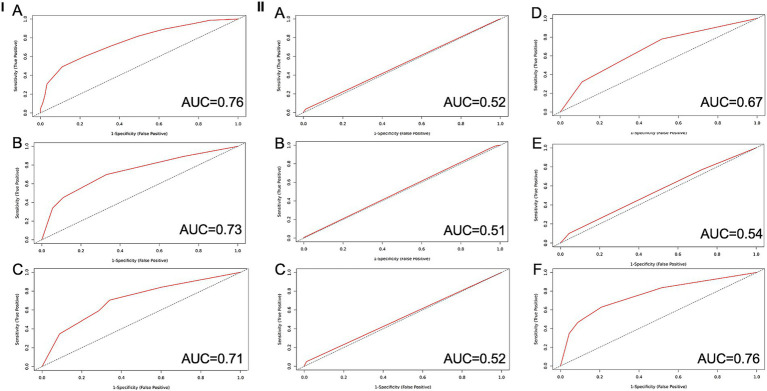
**I** ROC curves for the **A** SOFA score (AUC = 0.65, CI97.5% = 0.56–0.75), the **B** HH score (AUC = 0.64, CI97.5% = 0.54–0.74) and **C** the WFNS (AUC = 0.62, CI97.5% = 0.52–0.72) score as a predictor for delayed cerebral infarctions. **II** ROC curves for every parameter of the SOFA score as predictor for delayed cerebral infarctions, AUC 0.66 (CI97.5% = 0.56–0.75). **A** Creatinine as predictive value for DCI (AUC = 0.52), **B** Bilirubin as predictive value for DCI (AUC = 0.51), **C** Number of thrombocytes as predictive value for DCI (AUC = 0.52), **D** MAP as predictive value for DCI (AUC = 0.67), **E** Horovitz quotient as predictive value for DCI (AUC = 0.54), **F** GCS as predictive value for DCI (AUC = 0.76). AUC, Area under the curve; GCS, Glasgow Coma Scale; MAP, Mean Arterial Pressure; SAH, Subarachnoidal hemorrhage; SOFA, Sequential Organ Failure Assessment Score.

A comprehensive evaluation of the predictive performance of each parameter is presented in the following section and detailed in Figure 1. As shown in Figure 1, the three parameters — the Horovitz index, mean arterial pressure (MAP), and Glasgow Coma Scale (GCS) — performed best as individual predictors of outcome. These parameters are also the most relevant from a clinical perspective, as they reflect the organ systems most critically affected in this patient population.

The ICCTUS score had an AUC of 0.79 and an AIC of 282.6 to predict unfavorable outcome at 6 months (Figure 1A). In this case, the score achieved a sensitivity of 0.74 and a specificity of 0.74. The PPV was 0.83, and the NPV was 0.62. The Youden index was 0.48 and the cut-off was at 2 or higher points.

The most reliable parameter was the GCS, which reached an AUC equal to the AUC of the complete SOFA score (AUC = 0.76) and an AIC of 282.1 (Figure 1II,F). For the GCS alone, the sensitivity was 0.63 and the specificity was 0.79, whereas the PPV was 0.84 and the NPV was 0.55. The Youden index was 0.42 and the cut-off was 2.

The ICCTUS score, outperformed the other scores with respect to predictive accuracy and demonstrated the lowest AIC value, indicating the best overall model quality for the outcome parameter under evaluation (see below).

### Evaluation of the predictive value of the SOFA score and its individual parameters

To benchmark our score against an established reference, we also analyzed the predictive value of the full SOFA score for outcome after SAH. In addition, we assessed the prognostic contribution of each individual SOFA component, as well as relevant combinations of these parameters.

The Sequential Organ Failure Assessment (SOFA) score demonstrated the strongest predictive capacity for unfavorable outcome among all established scores. The optimal threshold was identified at a score ≥7. Patients with SOFA scores ≤6 exhibited a poor outcome in only 41.8% of cases, whereas 87.1% of patients with scores ≥7 developed an mRS ≥ 3 (*p* < 0.001). The Youden Index was 0.46, the highest among the assessed scores. The PPV was 0.42, and the NPV 0.42, sensitivity, and specificity were 0.46 and 0.11, respectively. The ROC curve for the SOFA score is displayed in Figure 1I,A, with an AUC of 0.76 and an AIC of 276.01. The identified cut-off of >6 points marks the threshold beyond which the risk of poor outcome substantially increases.

To reassess the rationale behind the selection of parameters included in the ICCTUS score and to evaluate their individual predictive value, each parameter of the SOFA score was examined in this context.

The parameter with the highest AUC was the GCS, with an AUC of 0.76 and an AIC of 282.05. It was followed by the MAP with an AUC of 0.67 and an AIC of 309.1. The third highest AUC value was the AUC of the Horovitz quotient with a value of 0.54 and an AIC of 332.74. The remaining two parameters are followed in descending order of their AUC values: bilirubin, creatinine level and thrombocytes. All AUC and AIC values and the consecutive graphs are shown in Figures 1II,A–F.

We compared the corresponding AUC values using the DeLong test to assess the differences in predictive performance between individual parameters. In summary, the parameter with the highest individual AUC value (GCS) significantly outperformed all the lower AUC values of the other parameters. The AUC of the Horovitz quotient was significantly better than the AUC of the MAP (*p* = 0.007). The MAP as the parameter with the third highest AUC outperformed Bilirubin, Creatinine and the number of thrombocytes (*p* < 0.001 for all comparisons).

### Evaluation of the predictive value of the Hunt and Hess score and WFNS score

In order to compare the ICCTUS score with existing prognostic tools for outcome prediction following subarachnoid hemorrhage, we assessed the predictive performance of both the Hunt and Hess score and the WFNS grading system.

The optimal threshold of the HH score associated with a significantly increased risk of unfavorable outcome was identified at grade 3. Among patients with an HH score below 3, 38.4% developed a poor functional outcome (mRS ≥ 3), whereas this was the case in 79.0% of patients with scores ranging from 3 to 5 (*p* < 0.001). The Youden Index for this threshold was 0.42. The positive predictive value (PPV) was 0.38, while the negative predictive value (NPV) was 0.42, sensitivity and specificity were 0.30 and 0.29, respectively. The predictive performance of the HH score is illustrated in Figure 1I,B, showing an area under the receiver operating characteristic curve (AUC) of 0.73 and an Akaike Information Criterion (AIC) of 288.86.

For the WFNS score, the optimal threshold predictive of a poor outcome was determined to be ≥3. In patients with WFNS grades 1–2, 42.0% experienced an unfavorable outcome, compared to 77.7% with WFNS grades ≥3 (*p* < 0.001). The PPV was 0.38, and the NPV was 0.42, with a sensitivity of 0.15 and a specificity of 0.58, resulting in a Youden Index of 0.38. The ROC analysis for WFNS score prediction is shown in Figure 1I,C, with an AUC of 0.71 and an AIC of 297.86.

### Comparison of the ICCTUS score, the SOFA score, the HH score and the WFNS score

In direct comparison, ROC analysis yielded AUCs of 0.71 for the WFNS score, 0.73 for the HH score, and 0.76 for the SOFA score, with the latter demonstrating the highest discriminatory power for predicting unfavorable functional outcome among the established scores. The DeLong test showed no significant difference between the AUC values of the three evaluated scores. The ROC analysis for these three scores is shown in Figure 1I.

This analysis revealed that the GCS as a one-parameter score was more reliable in predicting the outcome than all the other parameters of the SOFA score and the SOFA score in its complete form. There was no superiority compared to the HH score (*p* = 0.219) and only a trend toward a better predictive value concerning the WFNS score (*p* = 0.053).

Our newly developed ICCTUS score outperformed the established scoring systems, namely H&H and WFNS scores. This was reflected by a lower Akaike Information Criterion (AIC), indicating superior model performance in predicting clinical outcomes. The score performance was similar in comparison to the complete SOFA score. However, the ICCTUS score is simpler to evaluate and does not require extensive blood samples.

**Figure 2:**
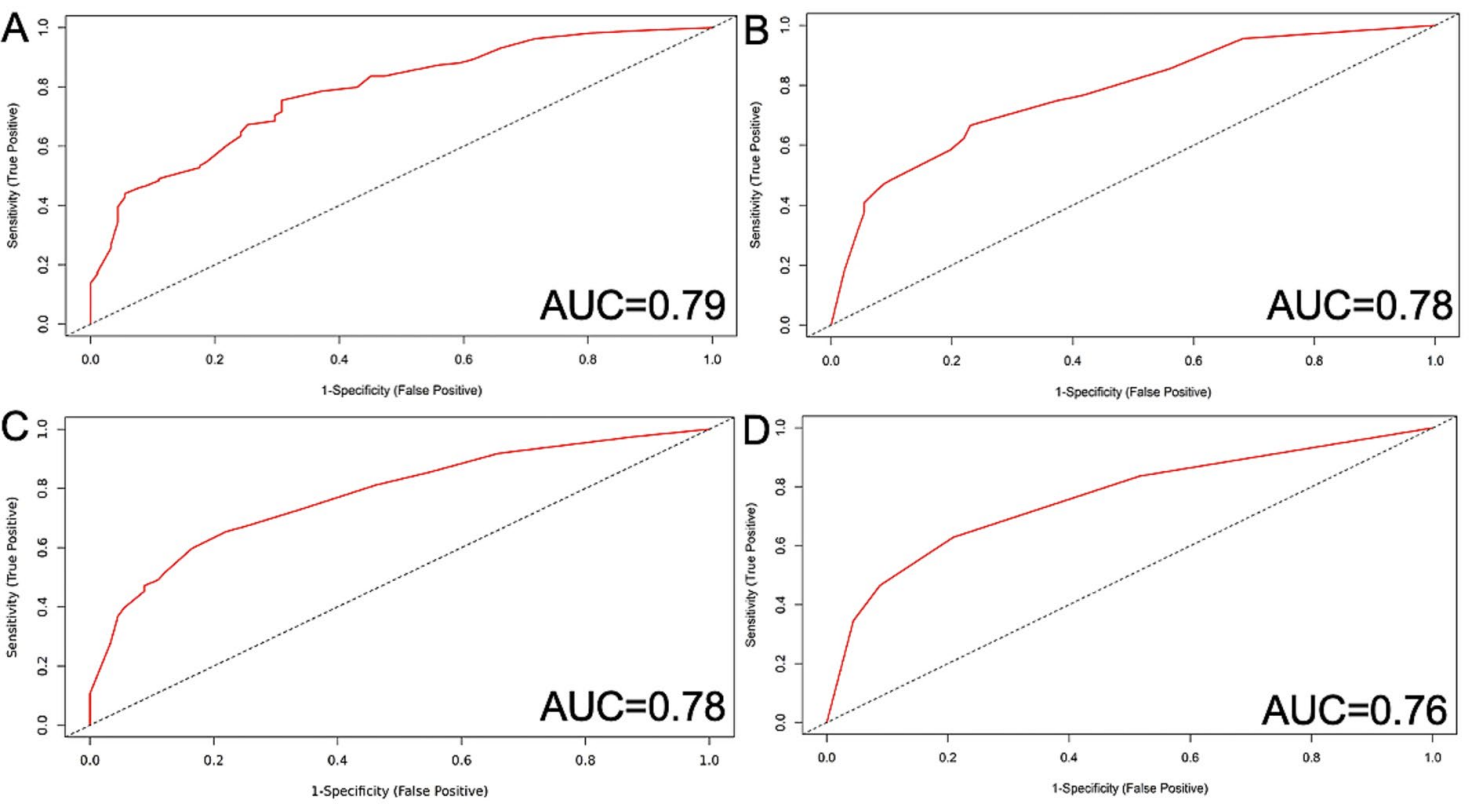
SOFA score parameters with the most robust predictive value for the outcome after SAH. **(A)** ICCTUS score: MAP, Horovitz quotient and GCS included, AUC = 0.79, **(B)** MAP and GCS, AUC = 0.78, **(C)** MAP and Horovitz quotient, AUC = 0.78, **(D)** GCS alone, AUC = 0.76. II Evaluation of specific risk factors and physiological parameters with regard to the clinical outcome following subarachnoid hemorrhage. AUC, Area under the curve; GCS, Glasgow Coma Scale; EVD, External Ventricular Drainage; HH, Hunt and Hess score; MAP, Mean Arterial Pressure; WFNS, Word Federation of Neurosurgical Societies; SAH, Subarachnoidal hemorrhage; SOFA, score Sequential Organ Failure Assessment Score.

Moreover, the ICCTUS score showed a predictive value presented by the area under the curve, which was significantly higher than the predictive value of the HH and the WFNS score (DeLong test: *p* = 0.02 and 0.003 respectively). Additionally, it tended to be superior to the complete SOFA score in the DeLong test, confirming improved discriminative ability (*p* = 0.061) (Figure 2).

## Discussion

In this study we analyzed the ability of a new score, the ICCTUS score, to predict unfavorable outcome after SAH. The ICCTUS score is a novel score derived from the SOFA score, only focusing on GCS, cardiac and pulmonary function. In our cohort the ICCTUS score performed better with regard of the predictive power for patient outcomes at 6 months following SAH compared to the commonly used HH, WFNS, and Fisher as well as the SOFA score. The score proved effective, achieving an AUC of 0.79 and an AIC of 237.0.

To assess the severity of SAH (20) and predict the risk of unfavorable outcome after the bleeding event, scores such as the HH, WFNS, and Fisher scores are commonly used. These scores are based on the neurological status, which reflects the degree of primary brain injury, or on the extent of intracranial blood clots. However, they do not account for peripheral organ dysfunctions, which are common conditions in the acute phase after SAH and can have significant impact on patient outcome (11, 24).

The most common peripheral organ dysfunction after SAH Is neurogenic cardiomyopathy (Lee, 2006 #3). It occurs at different levels of severity, ranging from ECG abnormalities to severe cardiac insufficiency, which can be associated with neurogenic pulmonary edema. Both, neurogenic cardiomyopathy and neurogenic pulmonary edema, can contribute to brain injury, by impairing cerebral blood and oxygen supply (Friedman, 2003 #4). The ICCTUS score combines the GCS, which early after SAH reflects the degree of EBI, with circulatory and pulmonary function, which in the context of SAH reflect the degree of neurogenic cardiomyopathy and neurogenic pulmonary edema. It is therefore plausible that the ICCTUS score is well suitable to predict outcome after SAH. The ICCTUS score outperformed the established scoring systems, namely the H&H and WFNS scores. The score performance was similar in comparison to the complete SOFA score. However, the ICCTUS score is simpler to evaluate and does not require extensive blood samples.

The SOFA score, which is routinely used in intensive care medicine, reflects a broad range of organ dysfunctions. It is, therefore, a reasonable conclusion that the SOFA score or at least assessment of cardiac and pulmonary function could be utilized for predicting outcomes after SAH (25, 26). In line with other studies (1, 15, 23) we observed that the SOFA score also predicted unfavorable outcome after SAH with similar predictive values as the H&H and WFNS scores. However, the SOFA score includes several organ dysfunctions, such as the coagulation system, and the liver and renal function, which are rarely affected after SAH.

Additionally, in a previous study it was demonstrated that the combination of MAP, Horovitz index, and GCS, operationalized as the ICCTUS score, can predict the development of DCI-associated infarctions. This represents a key driver of secondary brain injury after subarachnoid hemorrhage and thus an important determinant of functional outcome. Given that the score is also predictive in this context, it captures a crucial aspect of secondary brain damage and its impact on functional outcome after subarachnoid hemorrhage (19).

The ICCTUS score can be derived from routine documentation typically available in patients under ICU or IMC surveillance, without requiring additional procedures or data collection. This makes it well-suited for clinical implementation.

Early risk stratification for poor functional outcome or in-hospital mortality is essential to enable timely escalation of intensive care and interventional therapies—particularly in the context of neuroprotective treatment strategies. The ICCTUS score could be used as an additional instrument in the multimodal approach to assess the risk. Given the invasive nature and limited availability of advanced neuromonitoring techniques (e.g., parenchymal probes, repeated angiography), their use must be targeted toward patients who have a high risk of unfavorable outcome and who are most likely to benefit. Such a focused approach is not only justified from a resource management perspective but may also improve the overall quality of care and clinical outcomes. Comprehensive monitoring should therefore be concentrated on high-risk patients, and tools that enable such differentiation are of high practical relevance.

Naturally, prospective studies are needed to validate the score’s predictive performance and confirm its generalizability. Moreover, it should be viewed not as a replacement for established risk scores, but as an additional tool to improve early detection of patients at risk when used in conjunction with existing clinical assessments.

## Conclusion

Neurogenic cardiomyopathy and neurogenic pulmonary edema are common complications following subarachnoid hemorrhage (20) (Lee, 2006 #3), which, in addition to the primary neurological injury, significantly affect clinical outcomes.

Conventional prognostic scores, such as the Hunt and Hess and the World Federation of Neurological Surgeons scores, do not account for dysfunction in organ systems beyond the central nervous system (27) itself.

We developed the ICCTUS score to predict outcomes based on the most commonly affected organ systems after SAH. It incorporates mean arterial pressure, the Horovitz ratio (PaO₂/FiO₂), and the Glasgow Coma Scale, reflecting the functional status of the cardiovascular, pulmonary, and neurological systems, respectively. These parameters are already established components of the SOFA score for assessing organ function in critical care.

The predictive accuracy for the outcome after SAH of the ICCTUS score surpasses that of both the HH and WFNS scores and similar to SOFA score. To verify the predictive value of our score, external validation is planned. Nevertheless, it can be noted that, due to its reliance on routinely collected parameters in intensive care settings, the score is easy to apply and can be seamlessly integrated into clinical practice to improve outcome prediction following SAH.

### Limitations

There are some limitations to this study. Firstly, the retrospective character of the study can cause a selection bias, and our study is a single-center evaluation. The generalizability of the results is limited as the results are based on a cohort of patients treated in the same environment and with the same treatment protocol. Additionally, there, the patients’ collective was rather heterogeneous because of all grades of hemorrhages and different kinds of endovascular surgical treatment of aneurysms.

The score was developed using our complete cohort. No internal validation was performed, as the entire cohort was used for its development. An external validation in a multicenter cohort will be required to accurately assess the predictive value of the score and to determine the risk of poor outcome after SAH associated with each score point. This is our future goal.

## Data Availability

The original contributions presented in the study are included in the article/supplementary material, further inquiries can be directed to the corresponding authors.

## References

[1] Basile-Filho A Lago AF Menegueti MG Nicolini EA Nunes RS Lima SL . The use of SAPS 3, SOFA, and Glasgow coma scale to predict mortality in patients with subarachnoid hemorrhage: a retrospective cohort study. Medicine. (2018) 97:e12769. doi: 10.1097/MD.0000000000012769, 30313090 PMC6203557

[2] Claassen J Park S. Spontaneous subarachnoid haemorrhage. Lancet. (2022) 400:846–62. doi: 10.1016/S0140-6736(22)00938-2 35985353 PMC9987649

[3] Macdonald RL Schweizer TA. Spontaneous subarachnoid haemorrhage. Lancet. (2017) 389:655–66. doi: 10.1016/S0140-6736(16)30668-7, 27637674

[4] Macdonald RL. Delayed neurological deterioration after subarachnoid haemorrhage. Nat Rev Neurol. (2014) 10:44–58. doi: 10.1038/nrneurol.2013.246, 24323051

[5] Macdonald RL Pluta RM Zhang JH. Cerebral vasospasm after subarachnoid hemorrhage: the emerging revolution. Nat Clin Pract Neurol. (2007) 3:256–63. doi: 10.1038/ncpneuro0490 17479073

[6] Ikram A Javaid MA Ortega-Gutierrez S Selim M Kelangi S Anwar SMH . Delayed cerebral ischemia after subarachnoid hemorrhage. J Stroke Cerebrovasc Dis. (2021) 30:106064. doi: 10.1016/j.jstrokecerebrovasdis.2021.106064, 34464924

[7] Alsbrook DL Di Napoli M Bhatia K Desai M Hinduja A Rubinos CA . Pathophysiology of early brain injury and its association with delayed cerebral ischemia in aneurysmal subarachnoid hemorrhage: a review of current literature. J Clin Med. (2023) 12:1015. doi: 10.3390/jcm12031015, 36769660 PMC9918117

[8] Hunt WE Hess RM. Surgical risk as related to time of intervention in the repair of intracranial aneurysms. J Neurosurg. (1968) 28:14–20. 5635959 10.3171/jns.1968.28.1.0014

[9] Cavanagh SJ Gordon VL. Grading scales used in the management of aneurysmal subarachnoid hemorrhage: a critical review. J Neurosci Nurs. (2002) 34:288–95. doi: 10.1097/01376517-200212000-00002, 12506811

[10] Lee VH Oh JK Mulvagh SL Wijdicks EF. Mechanisms in neurogenic stress cardiomyopathy after aneurysmal subarachnoid hemorrhage. Neurocrit Care. (2006) 5:243–9. doi: 10.1385/NCC:5:3:243 17290097

[11] Friedman JA Pichelmann MA Piepgras DG McIver JI Toussaint LG McClelland RL . Pulmonary complications of aneurysmal subarachnoid hemorrhage. Neurosurgery. (2003) 52:1025–31. doi: 10.1093/neurosurgery/52.5.1025 12699543

[12] Murthy SB Shah S Rao CP Bershad EM Suarez JI. Neurogenic stunned myocardium following acute subarachnoid Hemorrhage: pathophysiology and practical considerations. J Intensive Care Med. (2015) 30:318–25. doi: 10.1177/0885066613511054, 24212600

[13] Kerro A Woods T Chang JJ. Neurogenic stunned myocardium in subarachnoid hemorrhage. J Crit Care. (2017) 38:27–34. doi: 10.1016/j.jcrc.2016.10.010, 27837689

[14] Temes RE Tessitore E Schmidt JM Naidech AM Fernandez A Ostapkovich ND . Left ventricular dysfunction and cerebral infarction from vasospasm after subarachnoid hemorrhage. Neurocrit Care. (2010) 13:359–65. doi: 10.1007/s12028-010-9447-x, 20945116

[15] Kurtz P Taccone FS Bozza FA Bastos LSL Righy C Gonçalves B . Systemic severity and organ dysfunction in subarachnoid Hemorrhage: a large retrospective Multicenter cohort study. Neurocrit Care. (2021) 35:56–61. doi: 10.1007/s12028-020-01139-3, 33150574

[16] Ferreira FL Bota DP Bross A Mélot C Vincent JL. Serial evaluation of the SOFA score to predict outcome in critically ill patients. JAMA. (2001) 286:1754–8. doi: 10.1001/jama.286.14.1754 11594901

[17] Lambden S Laterre PF Levy MM Francois B. The SOFA score-development, utility and challenges of accurate assessment in clinical trials. Crit Care. (2019) 23:374. doi: 10.1186/s13054-019-2663-7, 31775846 PMC6880479

[18] Vincent JL de Mendonça A Cantraine F Moreno R Takala J Suter PM . Use of the SOFA score to assess the incidence of organ dysfunction/failure in intensive care units: results of a multicenter, prospective study. Working group on "sepsis-related problems" of the European Society of Intensive Care Medicine. Crit Care Med. (1998) 26:1793–800. 9824069 10.1097/00003246-199811000-00016

[19] Kurz E Fassl V Brockmann C Schulze A Kalasauskas D Ringel F . Evaluation of the SOFA score as a tool to predict DCI-associated infarctions after spontaneous subarachnoid hemorrhage. Front Med. (2025) 12:1580643. doi: 10.3389/fmed.2025.1580643 PMC12229995 40625360

[20] Weyerbrock A Woznica M Rosahl SK Berlis A. Aneurysmal and non-aneurysmal SAH--is initial computed tomography predictive? Rofo. (2009) 181:881–7. doi: 10.1055/s-0028-1109355, 19401973

[21] Neulen A Pantel T König J Brockmann MA Ringel F Kantelhardt SR. Comparison of unruptured intracranial aneurysm treatment score and PHASES score in subarachnoid hemorrhage patients with multiple intracranial aneurysms. Front Neurol. (2021) 12:616497. doi: 10.3389/fneur.2021.616497 33897586 PMC8059702

[22] Neulen A Kunzelmann S Kosterhon M Pantel T Stein M Berres M . Automated grading of cerebral vasospasm to standardize computed tomography angiography examinations after subarachnoid hemorrhage. Front Neurol. (2020) 11:13. doi: 10.3389/fneur.2020.00013 32082241 PMC7002561

[23] Degrassi A Conticello C Njimi H Coppalini G Oliveira F Diosdado A . Grading scores for identifying patients at risk of delayed cerebral ischemia and neurological outcome in spontaneous subarachnoid hemorrhage: a comparison of receiver operator curve analysis. Neurocrit Care. (2025) 43:616–27. doi: 10.1007/s12028-025-02270-9 40293695

[24] Wartenberg KE Mayer SA. Medical complications after subarachnoid hemorrhage: new strategies for prevention and management. Curr Opin Crit Care. (2006) 12:78–84. doi: 10.1097/01.ccx.0000216571.80944.65, 16543780

[25] Chou SH. Inflammation, cerebral vasospasm, and brain injury in subarachnoid Hemorrhage-a shifting paradigm and a new beginning. Crit Care Med. (2018) 46:1883–5. doi: 10.1097/CCM.0000000000003373, 30312238 PMC6195224

[26] Carr KR Zuckerman SL Mocco J. Inflammation, cerebral vasospasm, and evolving theories of delayed cerebral ischemia. Neurol Res Int. (2013) 2013:506584. doi: 10.1155/2013/506584, 24058736 PMC3766617

[27] Kurz E Fassl V Brockmann C Schulze A Kalasauskas D Ringel F . Evaluation of the SOFA score as a tool to predict DCI-associated infarctions after spontaneous subarachnoid hemorrhage. Front Med (Lausanne). (2025) 12:1580643. doi: 10.3389/fmed.2025.1580643. Erratum in: Front Med (Lausanne). (2025) 12:1661506. doi: 10.3389/fmed.2025.1661506. 40625360; PMCID: PMC12229995. 40625360 PMC12229995

